# Civil-Military Coordination (CIMIC) Model in Natural Disasters in Iran

**DOI:** 10.30476/beat.2020.83646

**Published:** 2020-10

**Authors:** Hassan Araghizadeh, Mahmoudreza Peyravi, Simintaj Sharififar, Milad Ahmadi Marzaleh

**Affiliations:** 1 *Department of Anesthesiology, School of Medicine, Baghiyyatollah al-Azam Hospital, Baqiyatallah University of Medical sciences, Tehran, Iran*; 2 *Department of Health in Disasters and Emergencies, Health Human Resources Research Center, School of Management and Medical Informatics, Shiraz University of Medical Sciences, Shiraz, Iran*; 3 *Department of Health in Disasters and Emergencies, School of Nursing, AJA University of Medical Sciences, Tehran, Iran*; 4 *Research Center for Emergency and Disaster Resilience, Red Crescent society of the Islamic Republic of Iran, Tehran, Iran; *; 5 *Research Center for Health Management in Mass Gathering, Red Crescent Society of the Islamic Republic of Iran, Tehran, Iran*; 6 *Helal-Iran Institute, Red Crescent Society of the Islamic Republic of Iran, Tehran, Iran*; 7 *PhD of Health in Disasters and Emergencies, Student Research Committee, Department of Health in Disasters and Emergencies, Health Human Resources Research Center, School of Management and Medical Informatics, Shiraz University of Medical Sciences, Shiraz, Iran*; 8 *MPH of Health Policy, Health Policy Research Center, Institute of Health, Shiraz University of Medical Sciences, Fars, Iran*

**Keywords:** Coordination, Natural disasters, Military forces, Education, Civil, Iran

## Abstract

**Objective:**

The present study aimed at codifying a native model of civil-military coordination (CIMIC) in natural disasters in Iran.

**Methods:**

This manuscript is a part of a larger study. The present cross-sectional study was conducted in 2019 using a two-stage Delphi technique. The factors confirmed by the technique were prioritized via a pairwise questionnaire. In doing so, 24 elites and experts in civil-military coordination were presented with the indicators in the course of classic Delphi technique and pairwise comparison. At the end, the nationalized model was finalized by sending the model to ten experts and asking their ideas.

**Results:**

The results obtained from the two rounds of Delphi indicated that 36 coordination factors could be classified into three primary classes of staff, stuff, and system. All factors were confirmed by the experts. Considering the weight of each class, “staff” and “stuff” classes were considered to be the highest and lowest priorities, respectively.

**Conclusion:**

Application of the coordination factors in the context of military and civil organizations leads to a better response to natural disasters. The organizations in charge of responding to disasters should be obliged to apply this model in the highest organizational commitment level as the final goals of disaster management. The results of the present study can be applied for codification of a comprehensive plan for assessing the civil-military coordination in natural disasters.

## Introduction

Occurrence of various kinds of disasters has considerably increased during the 21^st^ century [1,2]. The people of developing countries are usually more intensively influenced by natural disasters and the majority of casualties also occur in these countries [3]. In the aftermath of disasters, the majority of civil organizations are afflicted with an inability to respond to accidents, so that they ask for contribution of military organizations [4]. Thus, it is necessary to make coordination between the military and civil actors in the course of responding to emergency conditions. Considering the daily increase in the occurrence of natural and man-made disasters, the presence of military forces in these disasters has been augmented [5]. However, there are problems including the discrepancy between the military and civil organizations that weaken their professional relationships. It is important for the military and civil organizations to have good relations for cooperation in the management of disasters [6]. In fact, governance of the inhomogeneous organizations that work together in disasters needs a novel approach to the central networking operation that can lead to information-sharing and coordination. In order to transcend beyond the normative and knowledge borders of the responding organizations, there is a need for consultation, sensitization, and dislocation of the structures [7]. Moreover, effective disaster management entails a fast and exact exchange of information [8]. The relationships between the international humanitarian organizations and army in providing relief was completely clear, but they predominantly worked in the form of separate and independent streams [9].

Iran is a disaster-prone country [10]. It is amongst the top ten disaster-prone countries and 90% of its population are exposed to the risk of earthquake and flood. In terms of natural disaster occurrence statistics, Iran has the sixth rank worldwide. Earthquake, flood, and drought are amongst the prevalent catastrophes in Iran [11]. So far, several deadly earthquakes have occurred in Iran, with Bam and Rudbar being the ones with the highest casualties [12]. If the activities of all organizations are managed in the preliminary stages after the disasters, few problems can arise. Generally, better coordination is detected among the organizations that commonly work with other organizations and promote and encourage these activities. According to the fact that Iran is a disaster-prone region and the military forces, including army, police, and Islamic Revolutionary Guards attend disasters for providing relief helps and response, it is highly important to have a nationalized model for enhancing coordination in natural disasters. Thus, the present study aims at codifying a customized civil-military coordination model in natural disasters in Iran in 2019.

## Materials and Methods

*Study Design*

This paper is a part of a larger study. The present cross-sectional research was carried out in five separate stages: 1) systematic review, 2) qualitative research, 3) Delphi technique, 4) Analytic Hierarchy Process (AHP), and 5) model delineation. After performing the systematic review and interviewing with Iranian experts, 36 factors that caused more coordination between the military and civil forces in the aftermath of disasters were extracted.

*Study Participants*

Classic Delphi technique was the method of choice for decision-making about the scales, which is used to determine the agreement of experts on an issue [13,14]. The indicators were presented in the first round of classic Delphi technique to 24 experts and specialists in crisis and disaster management and healthcare in disasters and emergencies and military men. The inclusion criteria were having at least a bachelor degree, having the experience of presence in disasters and/or managerial work on crisis and disaster management, having executive records and relevant researches, and being willing to participate in the study. If experts did not tend to participate in the study or did not have the mental preparation, they were not enrolled in the study.

*Data Collection and Data Analysis*

To perform the Delphi technique, we designed and distributed a questionnaire among the experts either by the researcher or via E-mail. The experts were asked to determine their importance rates based on a five-point Likert scale (1=very low importance, 2=low importance, 3=intermediate importance, 4=high importance, and 5=very high importance). Furthermore, the experts were asked to add other factors they thought as being important. The indicators with the mean scores below 2.5 were eliminated [15]. Other indicators were used in the second round in which those with the mean scores equal to or larger than three were confirmed and the rest (mean scores below three) were omitted.

In the case of agreement percentages above 75 out of a mean score of 5 (3.75) for each scale, the scales were accepted [16,17]. The cases with agreement percentages from 50 to 75 were used in the second round of Delphi technique that was administered to the same experts one month later. In various studies, there are discrepancies regarding the agreement threshold; however, the majority of experts consider 70-80% agreement as a sign of consensus achievement. Therefore, the agreement scale of the experts in the first round was a mean score above 75% [16,17]. At the end, the indicators of the identified civil-military coordination in natural disasters in Iran were finalized. The required information was obtained from each Delphi round based on statistical methods. Excel, version 2016 was used as well.

When the scales of the national model were specified and finalized in the course of Delphi technique, the ones with higher priorities were entered into the model. In this stage, the identified coordination indicators were extracted and utilized for codifying a pairwise questionnaire related to their prioritization and scoring. To prioritize the coordination indicators, use was made of AHP and Expert Choice Software, version 11. To perform scoring and determine the priority and significance of each of the indicators and sub-indicators pertinent to coordination, a pairwise comparison was made and the pairwise questionnaire was used in a range from +9 to -9.

AHP is one of the most comprehensive systems designed for decision making with multiple criteria which was introduced by Saaty for the first time [18]. Pairwise comparison is made based on the idea that how much element A is more important than element B [19]. The mean values were obtained for the ideas obtained from the 24 experts regarding each of the coordination factors; then, the analysis was commenced. In AHP, the elements of every level are compared to their counterparts from upper levels in a pairwise manner and their weights, called relative weights, are calculated. The relative weights are used to compute the final weight of each subclass. The final weight is obtained from multiplying the importance of each class by the weight of the subclass [20]. After performing the pairwise comparison, Expert Choice Software was employed. The acceptable inconsistency range in each system depends on the number of decision-makers. In general state, however, Saaty suggests that if the inconsistency of decision is more than 0.1, it is better for the decision-maker to change his/her judgment. For example, if the number of decision-makers is 10, the acceptable limit of inconsistency is 1.45. However, if the inconsistency coefficient is smaller than or equal to 0.1, the system is acceptable [18]. In addition, the combined weight is obtained through multiplying the weight of the scale by that of the subscale.

**Fig. 1:**
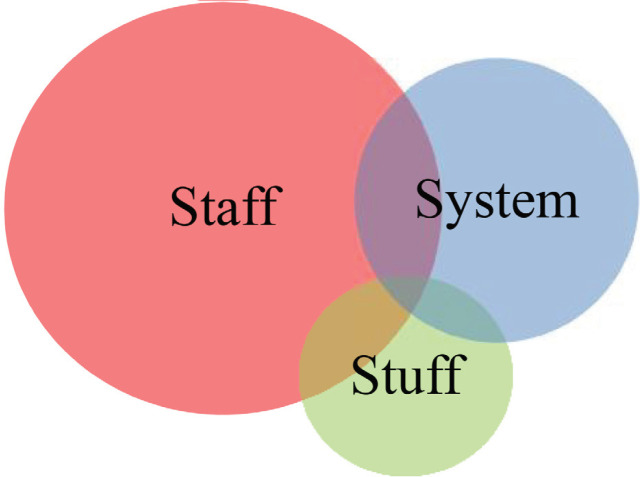
Model of civil-military coordination in natural disasters in Iran

Based on the previous stages, the researchers of the present study sent the model to ten experts and exchanged views with them regarding a national prototype of civil-military coordination in natural disasters in Iran (Figure 1). Eventually, the final model was designed, and its schematic view was drawn.

*Ethical considerations*

In the present study, the ethical considerations were as follows: 1) the experts’ written consent form, 2) willingness of the experts to accept or reject participation in the study, 3) confidentiality of the completed questionnaires and checklists, 4) acknowledgement and gratitude to the study participants, and 5) anonymity of all completed forms.

## Results

The mean age of the experts participating in the present study was 48.19± 6.9 years and all the experts were male. The demographic information and occupational specifications of the participants are shown in Table 1.

**Table 1:** Demographic and occupational characteristics of the contributors

**Frequency (%)**		**Characteristics**
24 (100)	Male	**Gender**
0 (0)	Female	
1 (4.1)	Bachelor	**Level of education**
3 (12.5)	Master of Sciences	
16 (66.6)	Doctor of Philosophy	
1 (4.1)	General Physician	
3 (12.)	Specialist Physician	
16 (66.6)	Civilian	**Field of expertise**
8 (33.3)	Military	

The results of the two rounds of Delphi technique implementation indicated that 36 coordination factors could be categorized into three primary classes, namely staff, stuff, and system. None of the factors was eliminated and all of them were confirmed by the experts.

In the first round, 31 factors were found with a high agreement level (75%) and five factors were found with an agreement percentage between 50 and 75. The factors with agreement percentages below 75 were presented to the experts in the second round of Delphi. At the end, all factors gained a high agreement level (75%) and none of them was omitted.

The extracted classes and subclasses were presented to the 24 experts, so that the priority of each factor could be determined in pairwise comparisons. The results obtained from the pairwise comparison and prioritization of the coordination factors are presented in Table 2. Considering the weight of each class, “staff” class had the highest priority and “stuff” class had the lowest priority. In the “staff” class, the subclass “knowledge and awareness” had the highest priority, and the subclass “creativity” had the lowest priority. In the system class, the subclasses “instruction and procedural unity” had the lowest priority. The “stuff” class only contained the subclass “novel communication technologies”.

**Table 2:** The prioritization of the final classes and subclasses of civil-military coordination in natural disasters

**Main class**	**Class weight**	**Priority**	**Subclass**	**Subclass weight**	**Combined weight**	**Priority**
Staff	0.623	1	Knowledge and awareness	0.1509	0.0940	1
			Trustable and obeyable commander	0.1432	0.0893	2
			Trust	0.1009	0.0628	3
			Common goal	0.0981	0.0611	4
			Experience	0.0811	0.0505	5
			Existence of a common language among individuals	0.0732	0.0456	6
			Presence of military men as commanders	0.0678	0.0422	7
			The forces’ self-sufficiency	0.0672	0.0418	8
			Work culture	0.0669	0.0416	9
			Criticism acceptance	0.0519	0.0323	10
			Avoidance of policies	0.0509	0.0317	11
			Creativity	0.0477	0.0297	12
System	0.325	2	Education	0.1534	0.0498	1
			Exercise	0.1522	0.0494	2
			Single commander	0.1490	0.0484	3
			Launching an incident command system (ICS)	0.0682	0.0221	4
			Rules, directions, guideline, protocols, and letters of agreement	0.0660	0.0214	5
			Holding common daily sessions	0.0587	0.0190	6
			Receiving and providing reports on a regular basis	0.0356	0.0115	7
			Having job description	0.0349	0.0113	8
			Formation of taskforces	0.0292	0.0094	9
			Transparency of the duties	0.0287	0.0093	10
			Information management	0.0265	0.0086	11
			Communications	0.0243	0.0078	12
			Task divisions	0.0233	0.0075	13
			Security	0.0210	0.0068	14
			Inter-organizational representative and link	0.0198	0.0064	15
			Safety	0.0178	0.0057	16
			Monitoring and control	0.0167	0.0054	17
			Standard operation procedures (SOPs)	0.0161	0.0052	18
			Planning	0.0154	0.0050	19
			Organizing activities	0.0143	0.0046	20
			Concentration on time	0.0128	0.0041	21
			Determination of the priorities	0.0093	0.0030	22
			Procedural unity	0.0068	0.0022	23
Stuff	0.052	3	Novel communication technologies	1	0.052	1

The consistency rates of all the studied cases were found to be below or equal to 0.1, which was acceptable.

After sending the preliminary model to ten experts via E-mail, based on the information obtained from the previous stages, the final model of civil-military coordination in natural disasters was attained as illustrated below. The size of each class and subclass was set based on their weights and priorities.

## Discussion

The present study aimed at codifying the civil-military coordination model in natural disasters in Iran. The coordination factors were categorized into three classes. The primary components of the model included staff, stuff, and system, each having some subclasses. After the investigations made by the study researchers, no model was found regarding the civil-military coordination in natural disasters in Iran and other countries. Various studies had only analyzed the factors influencing coordination separately and independently. The personnel are the most important component of coordination. The organizations should do their best in line with enhancing the knowledge and skill of their staff, so that the coordination among individuals can be elevated after the disasters. The staff comprise the most important piece for creating coordination in every organization and the resources would be wasted unless skillful human and managerial workforce are employed. In other words, the effectiveness, efficiency, and productivity of the staff are the most important principles and should be maximally taken into account.

The command structure and bureaucratic control play a top-down role in creating an integrated and united command bond between the army and numerous other organizations in disasters [21]. To precisely and rapidly transfer information, the authorities have to employ a unified commanding structure. The liaison officer plays an important role in connecting the officials in the organizations [22,23]. Effective coordination in response to emergency conditions necessitates information sharing, inter-personnel trust, and proper communication. Moreover, holding regular educational courses is effective in enhancement of the staff’s awareness and knowledge in the organizations [24, 25]. Participation in exercises can also bring about synergy between the military and civil workforce [25-27]. Indeed, application of an Incident Command System (ICS) causes more coordination among the staff from various organizations [27]. Various organizations fall in this structure and their activities become integrated in providing a unified response to an incident.

Civil-military coordination is of very great importance in disaster management. In natural disasters, military forces can act as a supportive structure and reduce the gaps and problems among civil forces [27]. Yet, assurance of the tasks fulfillment by the staff from the involved organizations in response to disasters is the responsibility of all the managers in organizations [25].

The use of military forces for supporting governments in response to disasters is quite common worldwide. However, each country follows a different approach in responding to these disasters. In response to Katrina Hurricane, there was political tension between the state and federal governments, which caused disorders in the responses. However, the civil commander cannot impose his/her orders to military commanders. Due to the same reason, it is suggested that military men should be appointed as the incident commanders. Furthermore, creation of an integrated command structure between the army and other involved organizations can enhance coordination [28]. Coordination systems should be launched before the occurrence of disasters in order to reduce overlapped and repetitive programs [29].

One of the study limitations was the inaccessibility of all experts and specialists for entering the Delphi technique and AHP. Thus, there was an attempt to employ the individuals who were rich in the required information. The model of civil-military coordination in natural disasters should be designed and validated for various communities, so that international organizations can make use of it for management plans and policymaking. The results of the present study can be applied for codification of a comprehensive plan for assessing the civil-military coordination in natural disasters.

The application of coordination factors in the context of military and civil organizations causes better response to natural disasters. The organizations proctoring response to disasters should be obliged in the highest level of organizational commitment to utilize this model, so that the final goals of disaster management, i.e. reduction of casualties, injuries, pains, and damages can be accomplished.

## References

[1] Myhre D, Bajaj S, Fehr L, Kapusta M, Woodley K, Nagji A (2017). Precepting at the time of a natural disaster. Clin Teach.

[2] Benjamin E, Bassily-Marcus AM, Babu E, Silver L, Martin ML (2011). Principles and practice of disaster relief: lessons from Haiti. Mt Sinai J Med.

[3] Wang X, Gao L, Shinfuku N, Zhang H, Zhao C, Shen Y (2000). Longitudinal study of earthquake-related PTSD in a randomly selected community sample in north China. Am J Psychiatry.

[4] Malešič M (2015). The impact of military engagement in disaster management on civil–military relations. Current sociology.

[5] Tatham P, Rietjens S (2016). Integrated disaster relief logistics: a stepping stone towards viable civil–military networks?. Disasters.

[6] Ibrahim N, Abdullah H, Roslan H (2018). Relationship between Civil and Military in Disaster Response and Recoverys. International Journal of Academic Research in Business and Social Sciences.

[7] Wolbers J (2016). Enhancing network centric operations doctrine to support civil military cooperation in disaster management. NL ARMS Netherlands Annual Review of Military Studies 2016: Springer;.

[8] Lichtenegger G, Vorraber W, Gojmerac I, Sporer A, Brugger J, Exner E (2015). Identification of information gaps in civil-military cooperation in disaster management. Information and Communication Technologies for Disaster Management (ICT-DM), 2015 2nd International Conference on.

[9] Arcala Hall R (2008). Civil-military cooperation in international disaster response: the Japanese Self-Defense Forces’ deployment in Aceh, Indonesia. The Korean Journal of Defense Analysis.

[10] Ghanbari V, Khankeh H, Hossaini M, Maddah S, Karimloo M, Ardalan A (2011). The effect of a disaster nursing education program on nurses’ preparedness for responding to probable natural disasters. Iran Journal of Nursing.

[11] Khankeh H (2007). Designing a Comprehensive Model for Health Disaster Management: Iran Medical Science University.

[12] Akbari ME, Farshad AA, Asadi-Lari M (2004). The devastation of Bam: an overview of health issues 1 month after the earthquake. Public Health.

[13] Powell C (2003). The Delphi technique: myths and realities. J Adv Nurs.

[14] Dempsey PA, Dempsey AD (2000). Using nursing research: Process, critical evaluation, and utilization.

[15] Karle H (2006). Global standards and accreditation in medical education: a view from the WFME. Acad Med.

[16] Hung H-L, Altschuld JW, Lee Y-F (2008). Methodological and conceptual issues confronting a cross-country Delphi study of educational program evaluation. Evaluation and program planning.

[17] Zeigler VL, Decker-Walters B (2010). Determining psychosocial research priorities for adolescents with implantable cardioverter defibrillators using Delphi methodology. J Cardiovasc Nurs.

[18] Saaty TL (2000). Fundamentals of decision making and priority theory.

[19] Lee AHI, Chen WC, Chang CJ (2008). A fuzzy AHP and BSC approach for evaluating performance of IT department in the manufacturing industry in Taiwan. Expert Systems with Applications.

[20] Ghodsi-pour SH (2007). Analytical hierarchy process (AHP).

[21] Berggren P, Nählinder S, Svensson E (2017). Characteristics of Command and Control in Response to Emergencies and Disasters Assessing Command and Control Effectiveness.

[22] Rietjens SJ, Verlaan K, Zaalberg TWB, De Boer SJ (2009). Inter‐organisational communication in civil–military cooperation during complex emergencies: a case study in Afghanistan 1. Disasters.

[23] Thompson WC (2010). Success in Kashmir: a positive trend in civil–military integration during humanitarian assistance operations. Disasters.

[24] McMaster R, Baber C (2012). Multi-agency operations: cooperation during flooding. Appl Ergon.

[25] Salmon P, Stanton N, Jenkins D, Walker G (2011). Coordination during multi-agency emergency response: issues and solutions. Disaster Prevention and Management: An International Journal.

[26] Nugroho SP, Pandanwangi TS, Suprapto S (2016). Civil-military cooperation in disaster management. Jurnal Pertahanan: Media Informasi ttg Kajian & Strategi Pertahanan yang Mengedepankan Identity, Nasionalism & Integrity.

[27] Scotter JRV, Pawlowski SD, Cu TD (2012). An examination of interdependencies among major barriers to coordination in disaster response. International Journal of Emergency Management.

[28] Burke R (2016). Lessons from Katrina: commanding the military during disaster response-then and now. International Journal of Emergency Management.

[29] Nabi PG (2014). Coordinating post-disaster humanitarian response: lessons from the 2005 Kashmir earthquake, India. Development in Practice.

